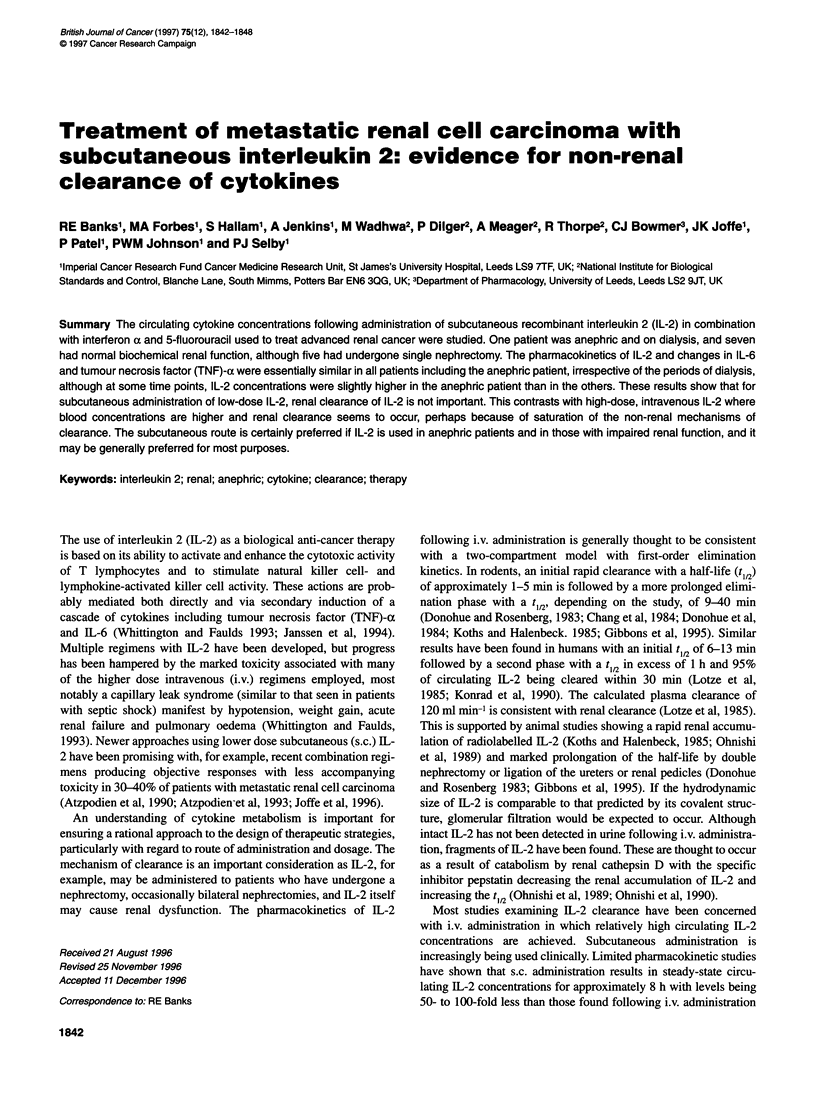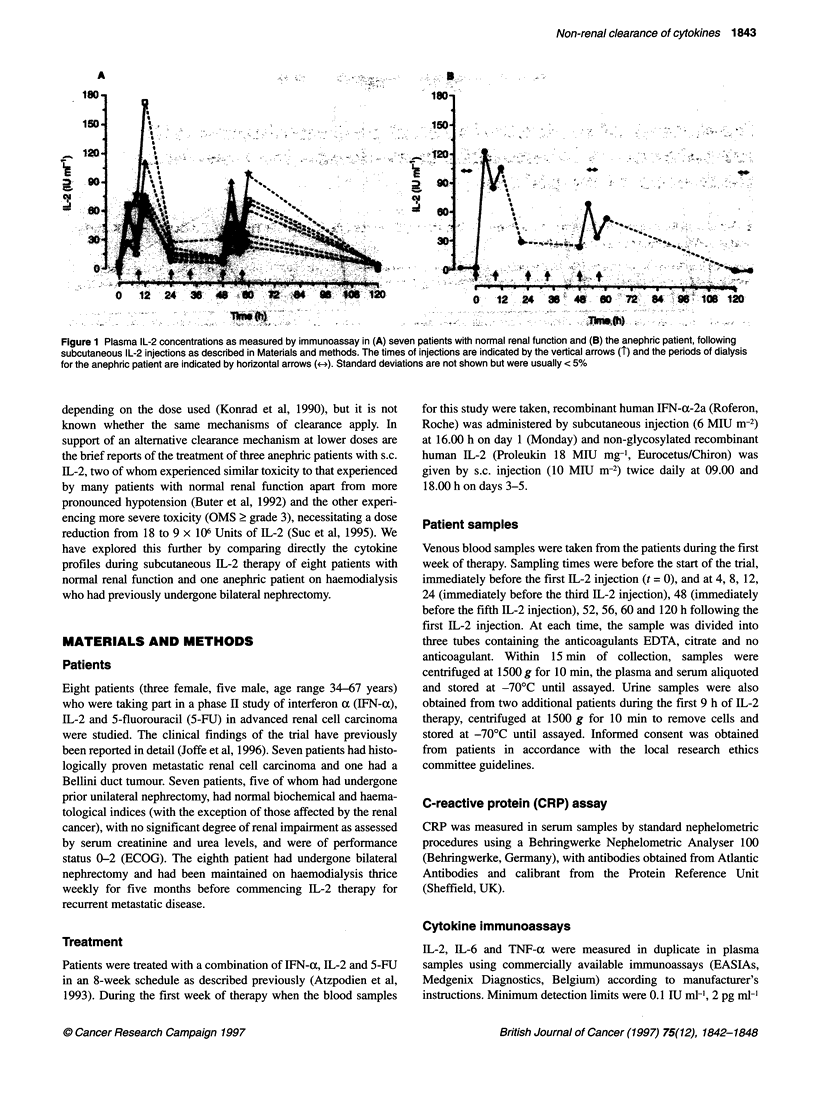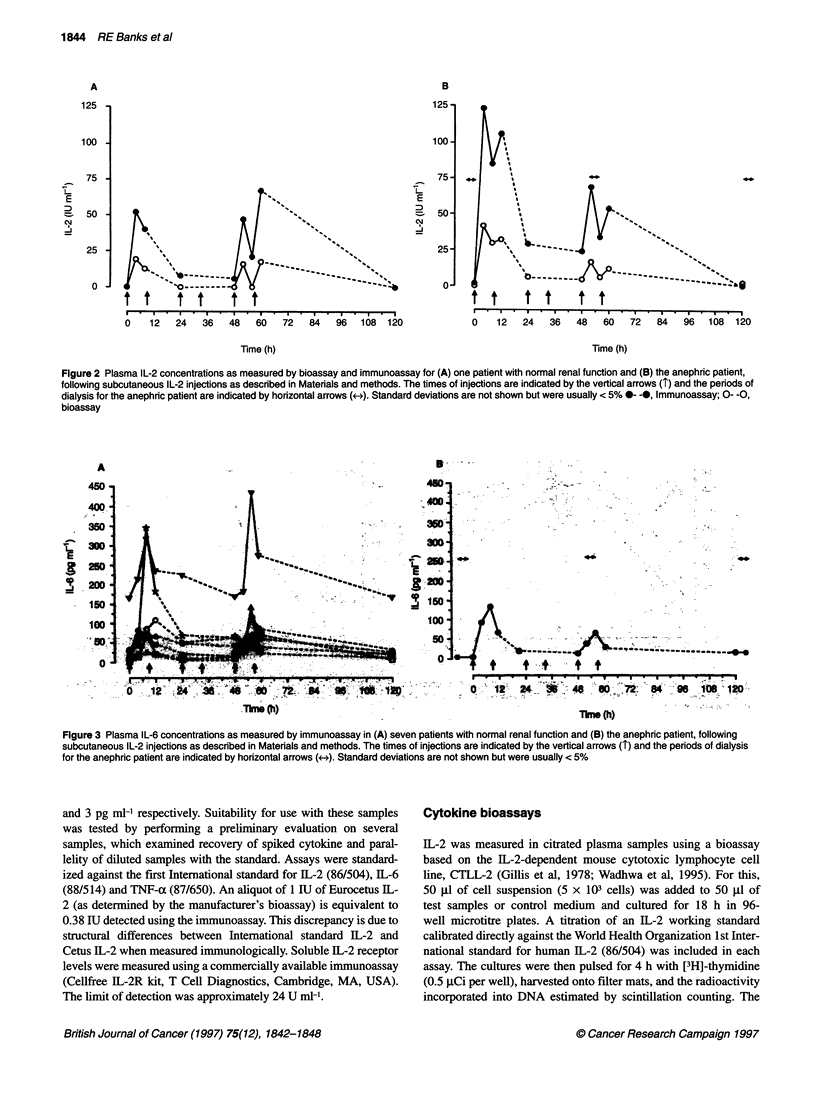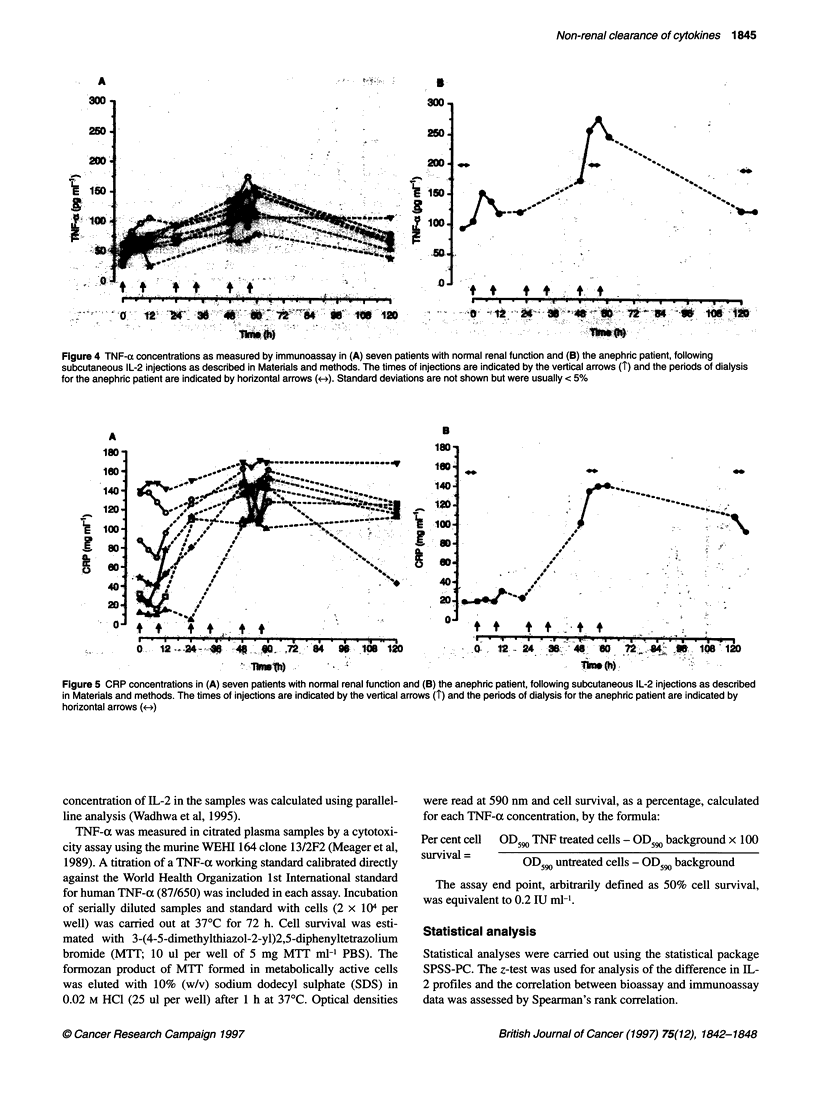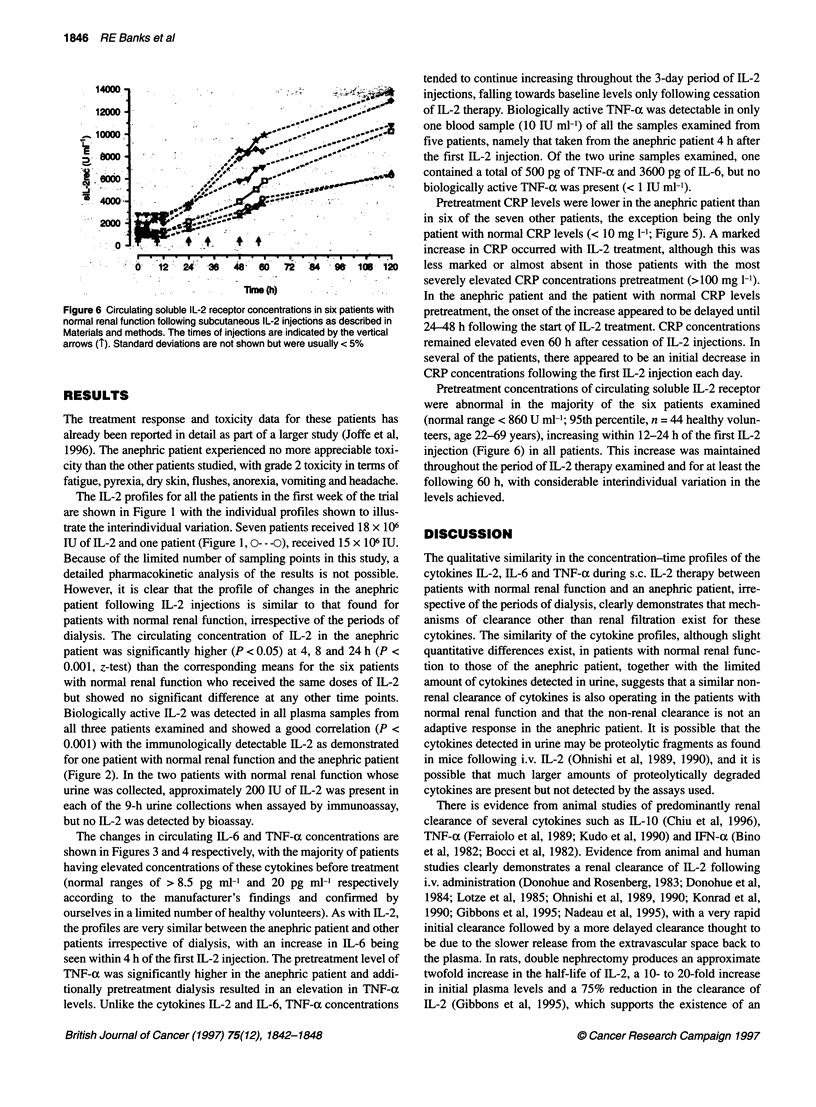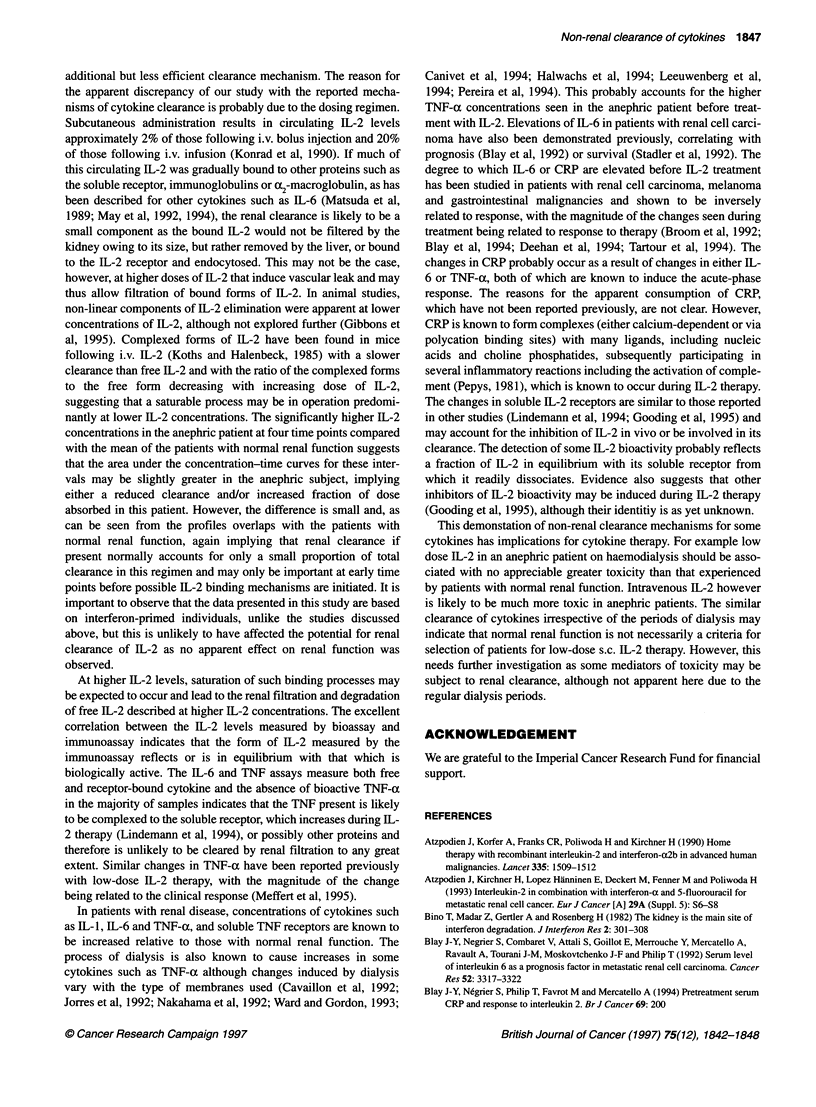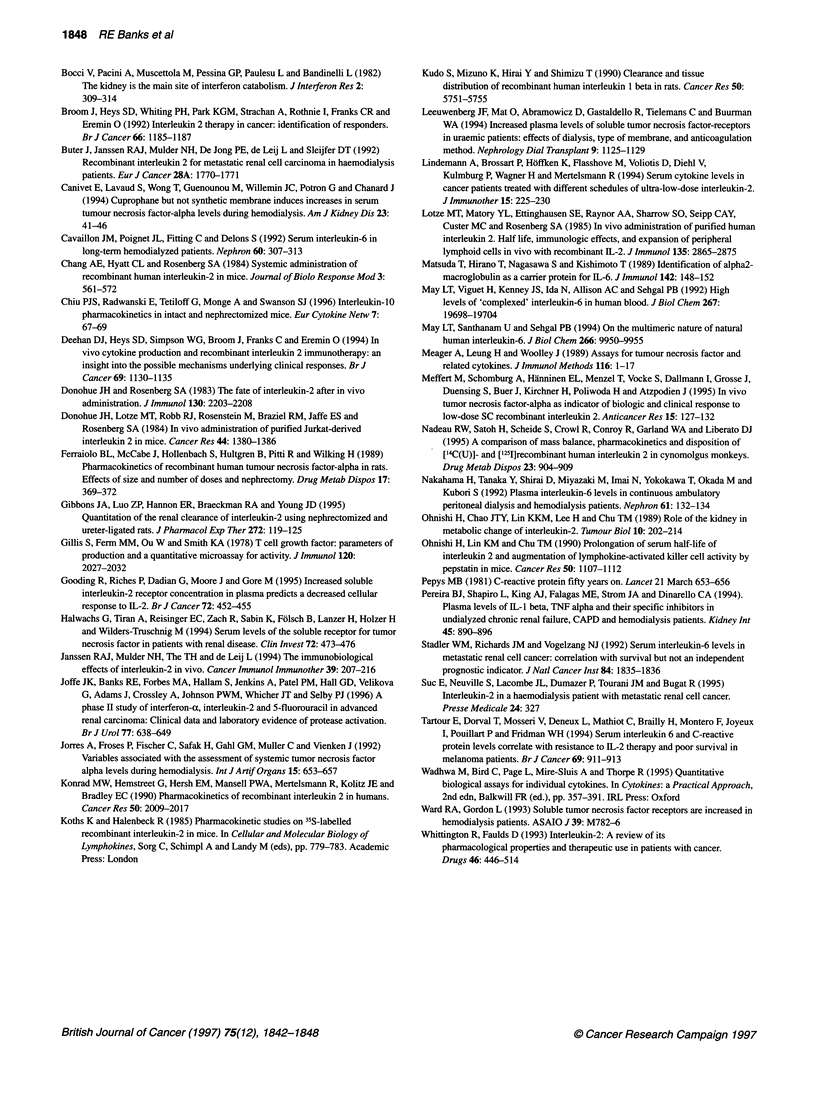# Treatment of metastatic renal cell carcinoma with subcutaneous interleukin 2: evidence for non-renal clearance of cytokines.

**DOI:** 10.1038/bjc.1997.314

**Published:** 1997

**Authors:** R. E. Banks, M. A. Forbes, S. Hallam, A. Jenkins, M. Wadhwa, P. Dilger, A. Meager, R. Thorpe, C. J. Bowmer, J. K. Joffe, P. Patel, P. W. Johnson, P. J. Selby

**Affiliations:** Imperial Cancer Research Fund Cancer Medicine Research Unit, St James's University Hospital, Leeds, UK.

## Abstract

The circulating cytokine concentrations following administration of subcutaneous recombinant interleukin 2 (IL-2) in combination with interferon alpha and 5-fluorouracil used to treat advanced renal cancer were studied. One patient was anephric and on dialysis, and seven had normal biochemical renal function, although five had undergone single nephrectomy. The pharmacokinetics of IL-2 and changes in IL-6 and tumour necrosis factor (TNF)-alpha were essentially similar in all patients including the anephric patient, irrespective of the periods of dialysis, although at some time points, IL-2 concentrations were slightly higher in the anephric patient than in the others. These results show that for subcutaneous administration of low-dose IL-2, renal clearance of IL-2 is not important. This contrasts with high-dose, intravenous IL-2 where blood concentrations are higher and renal clearance seems to occur, perhaps because of saturation of the non-renal mechanisms of clearance. The subcutaneous route is certainly preferred if IL-2 is used in anephric patients and in those with impaired renal function, and it may be generally preferred for most purposes.


British Journal of Cancer (1997) 75(12), 1842–1848
© 1997 Cancer Research Campaign

### Treatment of metastatic renal cell carcinoma with subcutaneous interleukin 2: evidence for non-renal clearance of cytokines

**RE Banks[1], MA Forbes[1], S Hallam[1], A Jenkins[1], M Wadhwa[2], P Dilger[2], A Meager[2], R Thorpe[2], CJ Bowmer[3], JK Joffe[1], P Patel[1], PWM Johnson[1] and PJ Selby[1]**

[1]Imperial Cancer Research Fund Cancer Medicine Research Unit, St James's University Hospital, Leeds LS9 7TF, UK; [2]National Institute for Biological Standards and Control, Blanche Lane, South Mimms, Potters Bar EN6 3QG, UK; [3]Department of Pharmacology, University of Leeds, Leeds LS2 9JT, UK

**Summary** The circulating cytokine concentrations following administration of subcutaneous recombinant interleukin 2 (IL-2) in combination with interferon α and 5-fluorouracil used to treat advanced renal cancer were studied. One patient was anephric and on dialysis, and seven had normal biochemical renal function, although five had undergone single nephrectomy. The pharmacokinetics of IL-2 and changes in IL-6 and tumour necrosis factor (TNF)-α were essentially similar in all patients including the anephric patient, irrespective of the periods of dialysis, although at some time points, IL-2 concentrations were slightly higher in the anephric patient than in the others. These results show that for subcutaneous administration of low-dose IL-2, renal clearance of IL-2 is not important. This contrasts with high-dose, intravenous IL-2 where blood concentrations are higher and renal clearance seems to occur, perhaps because of saturation of the non-renal mechanisms of clearance. The subcutaneous route is certainly preferred if IL-2 is used in anephric patients and in those with impaired renal function, and it may be generally preferred for most purposes.

**Keywords:** interleukin 2; renal; anephric; cytokine; clearance; therapy

The use of interleukin 2 (IL-2) as a biological anti-cancer therapy is based on its ability to activate and enhance the cytotoxic activity of T lymphocytes and to stimulate natural killer cell- and lymphokine-activated killer cell activity. These actions are probably mediated both directly and via secondary induction of a cascade of cytokines including tumour necrosis factor (TNF)-α and IL-6 (Whittington and Faulds 1993; Janssen et al, 1994). Multiple regimens with IL-2 have been developed, but progress has been hampered by the marked toxicity associated with many of the higher dose intravenous (i.v.) regimens employed, most notably a capillary leak syndrome (similar to that seen in patients with septic shock) manifest by hypotension, weight gain, acute renal failure and pulmonary oedema (Whittington and Faulds, 1993). Newer approaches using lower dose subcutaneous (s.c.) IL-2 have been promising with, for example, recent combination regimens producing objective responses with less accompanying toxicity in 30–40% of patients with metastatic renal cell carcinoma (Atzpodien et al, 1990; Atzpodien et al, 1993; Joffe et al, 1996).

An understanding of cytokine metabolism is important for ensuring a rational approach to the design of therapeutic strategies, particularly with regard to route of administration and dosage. The mechanism of clearance is an important consideration as IL-2, for example, may be administered to patients who have undergone a nephrectomy, occasionally bilateral nephrectomies, and IL-2 itself may cause renal dysfunction. The pharmacokinetics of IL-2 following i.v. administration is generally thought to be consistent with a two-compartment model with first-order elimination kinetics. In rodents, an initial rapid clearance with a half-life ($t_{1/2}$) of approximately 1–5 min is followed by a more prolonged elimination phase with a $t_{1/2}$, depending on the study, of 9–40 min (Donohue and Rosenberg, 1983; Chang et al, 1984; Donohue et al, 1984; Koths and Halenbeck. 1985; Gibbons et al, 1995). Similar results have been found in humans with an initial $t_{1/2}$ of 6–13 min followed by a second phase with a $t_{1/2}$ in excess of 1 h and 95% of circulating IL-2 being cleared within 30 min (Lotze et al, 1985; Konrad et al, 1990). The calculated plasma clearance of 120 ml $min^{-1}$ is consistent with renal clearance (Lotze et al, 1985). This is supported by animal studies showing a rapid renal accumulation of radiolabelled IL-2 (Koths and Halenbeck, 1985; Ohnishi et al, 1989) and marked prolongation of the half-life by double nephrectomy or ligation of the ureters or renal pedicles (Donohue and Rosenberg 1983; Gibbons et al, 1995). If the hydrodynamic size of IL-2 is comparable to that predicted by its covalent structure, glomerular filtration would be expected to occur. Although intact IL-2 has not been detected in urine following i.v. administration, fragments of IL-2 have been found. These are thought to occur as a result of catabolism by renal cathepsin D with the specific inhibitor pepstatin decreasing the renal accumulation of IL-2 and increasing the $t_{1/2}$ (Ohnishi et al, 1989; Ohnishi et al, 1990).

Most studies examining IL-2 clearance have been concerned with i.v. administration in which relatively high circulating IL-2 concentrations are achieved. Subcutaneous administration is increasingly being used clinically. Limited pharmacokinetic studies have shown that s.c. administration results in steady-state circulating IL-2 concentrations for approximately 8 h with levels being 50- to 100-fold less than those found following i.v. administration

*Received 21 August 1996*
*Revised 25 November 1996*
*Accepted 11 December 1996*

*Correspondence to:* RE Banks

**Figure 1** Plasma IL-2 concentrations as measured by immunoassay in (**A**) seven patients with normal renal function and (**B**) the anephric patient, following subcutaneous IL-2 injections as described in Materials and methods. The times of injections are indicated by the vertical arrows (↑) and the periods of dialysis for the anephric patient are indicated by horizontal arrows (↔). Standard deviations are not shown but were usually < 5%

depending on the dose used (Konrad et al, 1990), but it is not known whether the same mechanisms of clearance apply. In support of an alternative clearance mechanism at lower doses are the brief reports of the treatment of three anephric patients with s.c. IL-2, two of whom experienced similar toxicity to that experienced by many patients with normal renal function apart from more pronounced hypotension (Buter et al, 1992) and the other experiencing more severe toxicity (OMS ≥ grade 3), necessitating a dose reduction from 18 to 9 × $10^6$ Units of IL-2 (Suc et al, 1995). We have explored this further by comparing directly the cytokine profiles during subcutaneous IL-2 therapy of eight patients with normal renal function and one anephric patient on haemodialysis who had previously undergone bilateral nephrectomy.

### MATERIALS AND METHODS

### Patients

Eight patients (three female, five male, age range 34–67 years) who were taking part in a phase II study of interferon α (IFN-α), IL-2 and 5-fluorouracil (5-FU) in advanced renal cell carcinoma were studied. The clinical findings of the trial have previously been reported in detail (Joffe et al, 1996). Seven patients had histologically proven metastatic renal cell carcinoma and one had a Bellini duct tumour. Seven patients, five of whom had undergone prior unilateral nephrectomy, had normal biochemical and haematological indices (with the exception of those affected by the renal cancer), with no significant degree of renal impairment as assessed by serum creatinine and urea levels, and were of performance status 0–2 (ECOG). The eighth patient had undergone bilateral nephrectomy and had been maintained on haemodialysis thrice weekly for five months before commencing IL-2 therapy for recurrent metastatic disease.

### Treatment

Patients were treated with a combination of IFN-α, IL-2 and 5-FU in an 8-week schedule as described previously (Atzpodien et al, 1993). During the first week of therapy when the blood samples for this study were taken, recombinant human IFN-α-2a (Roferon, Roche) was administered by subcutaneous injection (6 MIU m$^{-2}$) at 16.00 h on day 1 (Monday) and non-glycosylated recombinant human IL-2 (Proleukin 18 MIU mg$^{-1}$, Eurocetus/Chiron) was given by s.c. injection (10 MIU m$^{-2}$) twice daily at 09.00 and 18.00 h on days 3–5.

### Patient samples

Venous blood samples were taken from the patients during the first week of therapy. Sampling times were before the start of the trial, immediately before the first IL-2 injection ($t$ = 0), and at 4, 8, 12, 24 (immediately before the third IL-2 injection), 48 (immediately before the fifth IL-2 injection), 52, 56, 60 and 120 h following the first IL-2 injection. At each time, the sample was divided into three tubes containing the anticoagulants EDTA, citrate and no anticoagulant. Within 15 min of collection, samples were centrifuged at 1500 ***g*** for 10 min, the plasma and serum aliquoted and stored at –70°C until assayed. Urine samples were also obtained from two additional patients during the first 9 h of IL-2 therapy, centrifuged at 1500 ***g*** for 10 min to remove cells and stored at –70°C until assayed. Informed consent was obtained from patients in accordance with the local research ethics committee guidelines.

### C-reactive protein (CRP) assay

CRP was measured in serum samples by standard nephelometric procedures using a Behringwerke Nephelometric Analyser 100 (Behringwerke, Germany), with antibodies obtained from Atlantic Antibodies and calibrant from the Protein Reference Unit (Sheffield, UK).

### Cytokine immunoassays

IL-2, IL-6 and TNF-α were measured in duplicate in plasma samples using commercially available immunoassays (EASIAs, Medgenix Diagnostics, Belgium) according to manufacturer's instructions. Minimum detection limits were 0.1 IU ml$^{-1}$, 2 pg ml$^{-1}$

© Cancer Research Campaign 1997

**Figure 2** Plasma IL-2 concentrations as measured by bioassay and immunoassay for (**A**) one patient with normal renal function and (**B**) the anephric patient, following subcutaneous IL-2 injections as described in Materials and methods. The times of injections are indicated by the vertical arrows (↑) and the periods of dialysis for the anephric patient are indicated by horizontal arrows (↔). Standard deviations are not shown but were usually < 5% ●- -●, Immunoassay; O- -O, bioassay

**Figure 3** Plasma IL-6 concentrations as measured by immunoassay in (**A**) seven patients with normal renal function and (**B**) the anephric patient, following subcutaneous IL-2 injections as described in Materials and methods. The times of injections are indicated by the vertical arrows (↑) and the periods of dialysis for the anephric patient are indicated by horizontal arrows (↔). Standard deviations are not shown but were usually < 5%

and 3 pg ml$^{-1}$ respectively. Suitability for use with these samples was tested by performing a preliminary evaluation on several samples, which examined recovery of spiked cytokine and parallelity of diluted samples with the standard. Assays were standardized against the first International standard for IL-2 (86/504), IL-6 (88/514) and TNF-α (87/650). An aliquot of 1 IU of Eurocetus IL-2 (as determined by the manufacturer's bioassay) is equivalent to 0.38 IU detected using the immunoassay. This discrepancy is due to structural differences between International standard IL-2 and Cetus IL-2 when measured immunologically. Soluble IL-2 receptor levels were measured using a commercially available immunoassay (Cellfree IL-2R kit, T Cell Diagnostics, Cambridge, MA, USA). The limit of detection was approximately 24 U ml$^{-1}$.

### Cytokine bioassays

IL-2 was measured in citrated plasma samples using a bioassay based on the IL-2-dependent mouse cytotoxic lymphocyte cell line, CTLL-2 (Gillis et al, 1978; Wadhwa et al, 1995). For this, 50 μl of cell suspension (5 × $10^3$ cells) was added to 50 μl of test samples or control medium and cultured for 18 h in 96-well microtitre plates. A titration of an IL-2 working standard calibrated directly against the World Health Organization 1st International standard for human IL-2 (86/504) was included in each assay. The cultures were then pulsed for 4 h with [$^3$H]-thymidine (0.5 μCi per well), harvested onto filter mats, and the radioactivity incorporated into DNA estimated by scintillation counting. The

 © Cancer Research Campaign 1997

**Figure 4** TNF-α concentrations as measured by immunoassay in (**A**) seven patients with normal renal function and (**B**) the anephric patient, following subcutaneous IL-2 injections as described in Materials and methods. The times of injections are indicated by the vertical arrows (↑) and the periods of dialysis for the anephric patient are indicated by horizontal arrows (↔). Standard deviations are not shown but were usually < 5%

**Figure 5** CRP concentrations in (**A**) seven patients with normal renal function and (**B**) the anephric patient, following subcutaneous IL-2 injections as described in Materials and methods. The times of injections are indicated by the vertical arrows (↑) and the periods of dialysis for the anephric patient are indicated by horizontal arrows (↔)

concentration of IL-2 in the samples was calculated using parallel-line analysis (Wadhwa et al, 1995).

TNF-α was measured in citrated plasma samples by a cytotoxicity assay using the murine WEHI 164 clone 13/2F2 (Meager et al, 1989). A titration of a TNF-α working standard calibrated directly against the World Health Organization 1st International standard for human TNF-α (87/650) was included in each assay. Incubation of serially diluted samples and standard with cells (2 × 10⁴ per well) was carried out at 37°C for 72 h. Cell survival was estimated with 3-(4-5-dimethylthiazol-2-yl)2,5-diphenyltetrazolium bromide (MTT; 10 ul per well of 5 mg MTT ml$^{-1}$ PBS). The formozan product of MTT formed in metabolically active cells was eluted with 10% (w/v) sodium dodecyl sulphate (SDS) in 0.02 M HCl (25 ul per well) after 1 h at 37°C. Optical densities were read at 590 nm and cell survival, as a percentage, calculated for each TNF-α concentration, by the formula:

$$\text{Per cent cell survival} = \frac{OD_{590}\ \text{TNF treated cells} - OD_{590}\ \text{background} \times 100}{OD_{590}\ \text{untreated cells} - OD_{590}\ \text{background}}$$

The assay end point, arbitrarily defined as 50% cell survival, was equivalent to 0.2 IU ml$^{-1}$.

### Statistical analysis

Statistical analyses were carried out using the statistical package SPSS-PC. The *z*-test was used for analysis of the difference in IL-2 profiles and the correlation between bioassay and immunoassay data was assessed by Spearman's rank correlation.

*© Cancer Research Campaign 1997* 

**Figure 6** Circulating soluble IL-2 receptor concentrations in six patients with normal renal function following subcutaneous IL-2 injections as described in Materials and methods. The times of injections are indicated by the vertical arrows (↑). Standard deviations are not shown but were usually < 5%

### RESULTS

The treatment response and toxicity data for these patients has already been reported in detail as part of a larger study (Joffe et al, 1996). The anephric patient experienced no more appreciable toxicity than the other patients studied, with grade 2 toxicity in terms of fatigue, pyrexia, dry skin, flushes, anorexia, vomiting and headache.

The IL-2 profiles for all the patients in the first week of the trial are shown in Figure 1 with the individual profiles shown to illustrate the interindividual variation. Seven patients received $18 \times 10^6$ IU of IL-2 and one patient (Figure 1, ○- - ○), received $15 \times 10^6$ IU. Because of the limited number of sampling points in this study, a detailed pharmacokinetic analysis of the results is not possible. However, it is clear that the profile of changes in the anephric patient following IL-2 injections is similar to that found for patients with normal renal function, irrespective of the periods of dialysis. The circulating concentration of IL-2 in the anephric patient was significantly higher ($P < 0.05$) at 4, 8 and 24 h ($P < 0.001$, $z$-test) than the corresponding means for the six patients with normal renal function who received the same doses of IL-2 but showed no significant difference at any other time points. Biologically active IL-2 was detected in all plasma samples from all three patients examined and showed a good correlation ($P < 0.001$) with the immunologically detectable IL-2 as demonstrated for one patient with normal renal function and the anephric patient (Figure 2). In the two patients with normal renal function whose urine was collected, approximately 200 IU of IL-2 was present in each of the 9-h urine collections when assayed by immunoassay, but no IL-2 was detected by bioassay.

The changes in circulating IL-6 and TNF-α concentrations are shown in Figures 3 and 4 respectively, with the majority of patients having elevated concentrations of these cytokines before treatment (normal ranges of > 8.5 pg ml$^{-1}$ and 20 pg ml$^{-1}$ respectively according to the manufacturer's findings and confirmed by ourselves in a limited number of healthy volunteers). As with IL-2, the profiles are very similar between the anephric patient and other patients irrespective of dialysis, with an increase in IL-6 being seen within 4 h of the first IL-2 injection. The pretreatment level of TNF-α was significantly higher in the anephric patient and additionally pretreatment dialysis resulted in an elevation in TNF-α levels. Unlike the cytokines IL-2 and IL-6, TNF-α concentrations tended to continue increasing throughout the 3-day period of IL-2 injections, falling towards baseline levels only following cessation of IL-2 therapy. Biologically active TNF-α was detectable in only one blood sample (10 IU ml$^{-1}$) of all the samples examined from five patients, namely that taken from the anephric patient 4 h after the first IL-2 injection. Of the two urine samples examined, one contained a total of 500 pg of TNF-α and 3600 pg of IL-6, but no biologically active TNF-α was present (< 1 IU ml$^{-1}$).

Pretreatment CRP levels were lower in the anephric patient than in six of the seven other patients, the exception being the only patient with normal CRP levels (< 10 mg l$^{-1}$; Figure 5). A marked increase in CRP occurred with IL-2 treatment, although this was less marked or almost absent in those patients with the most severely elevated CRP concentrations pretreatment (>100 mg l$^{-1}$). In the anephric patient and the patient with normal CRP levels pretreatment, the onset of the increase appeared to be delayed until 24–48 h following the start of IL-2 treatment. CRP concentrations remained elevated even 60 h after cessation of IL-2 injections. In several of the patients, there appeared to be an initial decrease in CRP concentrations following the first IL-2 injection each day.

Pretreatment concentrations of circulating soluble IL-2 receptor were abnormal in the majority of the six patients examined (normal range < 860 U ml$^{-1}$; 95th percentile, $n = 44$ healthy volunteers, age 22–69 years), increasing within 12–24 h of the first IL-2 injection (Figure 6) in all patients. This increase was maintained throughout the period of IL-2 therapy examined and for at least the following 60 h, with considerable interindividual variation in the levels achieved.

### DISCUSSION

The qualitative similarity in the concentration–time profiles of the cytokines IL-2, IL-6 and TNF-α during s.c. IL-2 therapy between patients with normal renal function and an anephric patient, irrespective of the periods of dialysis, clearly demonstrates that mechanisms of clearance other than renal filtration exist for these cytokines. The similarity of the cytokine profiles, although slight quantitative differences exist, in patients with normal renal function to those of the anephric patient, together with the limited amount of cytokines detected in urine, suggests that a similar non-renal clearance of cytokines is also operating in the patients with normal renal function and that the non-renal clearance is not an adaptive response in the anephric patient. It is possible that the cytokines detected in urine may be proteolytic fragments as found in mice following i.v. IL-2 (Ohnishi et al, 1989, 1990), and it is possible that much larger amounts of proteolytically degraded cytokines are present but not detected by the assays used.

There is evidence from animal studies of predominantly renal clearance of several cytokines such as IL-10 (Chiu et al, 1996), TNF-α (Ferraiolo et al, 1989; Kudo et al, 1990) and IFN-α (Bino et al, 1982; Bocci et al, 1982). Evidence from animal and human studies clearly demonstrates a renal clearance of IL-2 following i.v. administration (Donohue and Rosenberg, 1983; Donohue et al, 1984; Lotze et al, 1985; Ohnishi et al, 1989, 1990; Konrad et al, 1990; Gibbons et al, 1995; Nadeau et al, 1995), with a very rapid initial clearance followed by a more delayed clearance thought to be due to the slower release from the extravascular space back to the plasma. In rats, double nephrectomy produces an approximate twofold increase in the half-life of IL-2, a 10- to 20-fold increase in initial plasma levels and a 75% reduction in the clearance of IL-2 (Gibbons et al, 1995), which supports the existence of an

© Cancer Research Campaign 1997

additional but less efficient clearance mechanism. The reason for the apparent discrepancy of our study with the reported mechanisms of cytokine clearance is probably due to the dosing regimen. Subcutaneous administration results in circulating IL-2 levels approximately 2% of those following i.v. bolus injection and 20% of those following i.v. infusion (Konrad et al, 1990). If much of this circulating IL-2 was gradually bound to other proteins such as the soluble receptor, immunoglobulins or $\alpha_2$-macroglobulin, as has been described for other cytokines such as IL-6 (Matsuda et al, 1989; May et al, 1992, 1994), the renal clearance is likely to be a small component as the bound IL-2 would not be filtered by the kidney owing to its size, but rather removed by the liver, or bound to the IL-2 receptor and endocytosed. This may not be the case, however, at higher doses of IL-2 that induce vascular leak and may thus allow filtration of bound forms of IL-2. In animal studies, non-linear components of IL-2 elimination were apparent at lower concentrations of IL-2, although not explored further (Gibbons et al, 1995). Complexed forms of IL-2 have been found in mice following i.v. IL-2 (Koths and Halenbeck, 1985) with a slower clearance than free IL-2 and with the ratio of the complexed forms to the free form decreasing with increasing dose of IL-2, suggesting that a saturable process may be in operation predominantly at lower IL-2 concentrations. The significantly higher IL-2 concentrations in the anephric patient at four time points compared with the mean of the patients with normal renal function suggests that the area under the concentration–time curves for these intervals may be slightly greater in the anephric subject, implying either a reduced clearance and/or increased fraction of dose absorbed in this patient. However, the difference is small and, as can be seen from the profiles overlaps with the patients with normal renal function, again implying that renal clearance if present normally accounts for only a small proportion of total clearance in this regimen and may only be important at early time points before possible IL-2 binding mechanisms are initiated. It is important to observe that the data presented in this study are based on interferon-primed individuals, unlike the studies discussed above, but this is unlikely to have affected the potential for renal clearance of IL-2 as no apparent effect on renal function was observed.

At higher IL-2 levels, saturation of such binding processes may be expected to occur and lead to the renal filtration and degradation of free IL-2 described at higher IL-2 concentrations. The excellent correlation between the IL-2 levels measured by bioassay and immunoassay indicates that the form of IL-2 measured by the immunoassay reflects or is in equilibrium with that which is biologically active. The IL-6 and TNF assays measure both free and receptor-bound cytokine and the absence of bioactive TNF-α in the majority of samples indicates that the TNF present is likely to be complexed to the soluble receptor, which increases during IL-2 therapy (Lindemann et al, 1994), or possibly other proteins and therefore is unlikely to be cleared by renal filtration to any great extent. Similar changes in TNF-α have been reported previously with low-dose IL-2 therapy, with the magnitude of the change being related to the clinical response (Meffert et al, 1995).

In patients with renal disease, concentrations of cytokines such as IL-1, IL-6 and TNF-α, and soluble TNF receptors are known to be increased relative to those with normal renal function. The process of dialysis is also known to cause increases in some cytokines such as TNF-α although changes induced by dialysis vary with the type of membranes used (Cavaillon et al, 1992; Jorres et al, 1992; Nakahama et al, 1992; Ward and Gordon, 1993; Canivet et al, 1994; Halwachs et al, 1994; Leeuwenberg et al, 1994; Pereira et al, 1994). This probably accounts for the higher TNF-α concentrations seen in the anephric patient before treatment with IL-2. Elevations of IL-6 in patients with renal cell carcinoma have also been demonstrated previously, correlating with prognosis (Blay et al, 1992) or survival (Stadler et al, 1992). The degree to which IL-6 or CRP are elevated before IL-2 treatment has been studied in patients with renal cell carcinoma, melanoma and gastrointestinal malignancies and shown to be inversely related to response, with the magnitude of the changes seen during treatment being related to response to therapy (Broom et al, 1992; Blay et al, 1994; Deehan et al, 1994; Tartour et al, 1994). The changes in CRP probably occur as a result of changes in either IL-6 or TNF-α, both of which are known to induce the acute-phase response. The reasons for the apparent consumption of CRP, which have not been reported previously, are not clear. However, CRP is known to form complexes (either calcium-dependent or via polycation binding sites) with many ligands, including nucleic acids and choline phosphatides, subsequently participating in several inflammatory reactions including the activation of complement (Pepys, 1981), which is known to occur during IL-2 therapy. The changes in soluble IL-2 receptors are similar to those reported in other studies (Lindemann et al, 1994; Gooding et al, 1995) and may account for the inhibition of IL-2 in vivo or be involved in its clearance. The detection of some IL-2 bioactivity probably reflects a fraction of IL-2 in equilibrium with its soluble receptor from which it readily dissociates. Evidence also suggests that other inhibitors of IL-2 bioactivity may be induced during IL-2 therapy (Gooding et al, 1995), although their identitiy is as yet unknown.

This demonstation of non-renal clearance mechanisms for some cytokines has implications for cytokine therapy. For example low dose IL-2 in an anephric patient on haemodialysis should be associated with no appreciable greater toxicity than that experienced by patients with normal renal function. Intravenous IL-2 however is likely to be much more toxic in anephric patients. The similar clearance of cytokines irrespective of the periods of dialysis may indicate that normal renal function is not necessarily a criteria for selection of patients for low-dose s.c. IL-2 therapy. However, this needs further investigation as some mediators of toxicity may be subject to renal clearance, although not apparent here due to the regular dialysis periods.

### ACKNOWLEDGEMENT

We are grateful to the Imperial Cancer Research Fund for financial support.

### REFERENCES

Atzpodien J, Korfer A, Franks CR, Poliwoda H and Kirchner H (1990) Home therapy with recombinant interleukin-2 and interferon-α2b in advanced human malignancies. *Lancet* **335**: 1509–1512

Atzpodien J, Kirchner H, Lopez Hänninen E, Deckert M, Fenner M and Poliwoda H (1993) Interleukin-2 in combination with interferon-α and 5-fluorouracil for metastatic renal cell cancer. *Eur J Cancer* [A] **29A** (Suppl. 5): S6–S8

Bino T, Madar Z, Gertler A and Rosenberg H (1982) The kidney is the main site of interferon degradation. *J Interferon Res* **2**: 301–308

Blay J-Y, Negrier S, Combaret V, Attali S, Goillot E, Merrouche Y, Mercatello A, Ravault A, Tourani J-M, Moskovtchenko J-F and Philip T (1992) Serum level of interleukin 6 as a prognosis factor in metastatic renal cell carcinoma. *Cancer Res* **52**: 3317–3322

Blay J-Y, Négrier S, Philip T, Favrot M and Mercatello A (1994) Pretreatment serum CRP and response to interleukin 2. *Br J Cancer* **69**: 200

© Cancer Research Campaign 1997

Bocci V, Pacini A, Muscettola M, Pessina GP, Paulesu L and Bandinelli L (1982) The kidney is the main site of interferon catabolism. *J Interferon Res* **2**: 309–314

Broom J, Heys SD, Whiting PH, Park KGM, Strachan A, Rothnie I, Franks CR and Eremin O (1992) Interleukin 2 therapy in cancer: identification of responders. *Br J Cancer* **66**: 1185–1187

Buter J, Janssen RAJ, Mulder NH, De Jong PE, de Leij L and Sleijfer DT (1992) Recombinant interleukin 2 for metastatic renal cell carcinoma in haemodialysis patients. *Eur J Cancer* **28A**: 1770–1771

Canivet E, Lavaud S, Wong T, Guenounou M, Willemin JC, Potron G and Chanard J (1994) Cuprophane but not synthetic membrane induces increases in serum tumour necrosis factor-alpha levels during hemodialysis. *Am J Kidney Dis* **23**: 41–46

Cavaillon JM, Poignet JL, Fitting C and Delons S (1992) Serum interleukin-6 in long-term hemodialyzed patients. *Nephron* **60**: 307–313

Chang AE, Hyatt CL and Rosenberg SA (1984) Systemic administration of recombinant human interleukin-2 in mice. *Journal of Biolo Response Mod* **3**: 561–572

Chiu PJS, Radwanski E, Tetiloff G, Monge A and Swanson SJ (1996) Interleukin-10 pharmacokinetics in intact and nephrectomized mice. *Eur Cytokine Netw* **7**: 67–69

Deehan DJ, Heys SD, Simpson WG, Broom J, Franks C and Eremin O (1994) In vivo cytokine production and recombinant interleukin 2 immunotherapy: an insight into the possible mechanisms underlying clinical responses. *Br J Cancer* **69**: 1130–1135

Donohue JH and Rosenberg SA (1983) The fate of interleukin-2 after in vivo administration. *J Immunol* **130**: 2203–2208

Donohue JH, Lotze MT, Robb RJ, Rosenstein M, Braziel RM, Jaffe ES and Rosenberg SA (1984) In vivo administration of purified Jurkat-derived interleukin 2 in mice. *Cancer Res* **44**: 1380–1386

Ferraiolo BL, McCabe J, Hollenbach S, Hultgren B, Pitti R and Wilking H (1989) Pharmacokinetics of recombinant human tumour necrosis factor-alpha in rats. Effects of size and number of doses and nephrectomy. *Drug Metab Dispos* **17**: 369–372

Gibbons JA, Luo ZP, Hannon ER, Braeckman RA and Young JD (1995) Quantitation of the renal clearance of interleukin-2 using nephrectomized and ureter-ligated rats. *J Pharmacol Exp Ther* **272**: 119–125

Gillis S, Ferm MM, Ou W and Smith KA (1978) T cell growth factor: parameters of production and a quantitative microassay for activity. *J Immunol* **120**: 2027–2032

Gooding R, Riches P, Dadian G, Moore J and Gore M (1995) Increased soluble interleukin-2 receptor concentration in plasma predicts a decreased cellular response to IL-2. *Br J Cancer* **72**: 452–455

Halwachs G, Tiran A, Reisinger EC, Zach R, Sabin K, Fölsch B, Lanzer H, Holzer H and Wilders-Truschnig M (1994) Serum levels of the soluble receptor for tumor necrosis factor in patients with renal disease. *Clin Invest* **72**: 473–476

Janssen RAJ, Mulder NH, The TH and de Leij L (1994) The immunobiological effects of interleukin-2 in vivo. *Cancer Immunol Immunother* **39**: 207–216

Joffe JK, Banks RE, Forbes MA, Hallam S, Jenkins A, Patel PM, Hall GD, Velikova G, Adams J, Crossley A, Johnson PWM, Whicher JT and Selby PJ (1996) A phase II study of interferon-α, interleukin-2 and 5-fluorouracil in advanced renal carcinoma: Clinical data and laboratory evidence of protease activation. *Br J Urol* **77**: 638–649

Jorres A, Froses P, Fischer C, Safak H, Gahl GM, Muller C and Vienken J (1992) Variables associated with the assessment of systemic tumor necrosis factor alpha levels during hemodialysis. *Int J Artif Organs* **15**: 653–657

Konrad MW, Hemstreet G, Hersh EM, Mansell PWA, Mertelsmann R, Kolitz JE and Bradley EC (1990) Pharmacokinetics of recombinant interleukin 2 in humans. *Cancer Res* **50**: 2009–2017

Koths K and Halenbeck R (1985) Pharmacokinetic studies on $^{35}S$-labelled recombinant interleukin-2 in mice. In *Cellular and Molecular Biology of Lymphokines*, Sorg C, Schimpl A and Landy M (eds), pp. 779–783. Academic Press: London

Kudo S, Mizuno K, Hirai Y and Shimizu T (1990) Clearance and tissue distribution of recombinant human interleukin 1 beta in rats. *Cancer Res* **50**: 5751–5755

Leeuwenberg JF, Mat O, Abramowicz D, Gastaldello R, Tielemans C and Buurman WA (1994) Increased plasma levels of soluble tumor necrosis factor-receptors in uraemic patients: effects of dialysis, type of membrane, and anticoagulation method. *Nephrology Dial Transplant* **9**: 1125–1129

Lindemann A, Brossart P, Höffken K, Flasshove M, Voliotis D, Diehl V, Kulmburg P, Wagner H and Mertelsmann R (1994) Serum cytokine levels in cancer patients treated with different schedules of ultra-low-dose interleukin-2. *J Immunother* **15**: 225–230

Lotze MT, Matory YL, Ettinghausen SE, Raynor AA, Sharrow SO, Seipp CAY, Custer MC and Rosenberg SA (1985) In vivo administration of purified human interleukin 2. Half life, immunologic effects, and expansion of peripheral lymphoid cells in vivo with recombinant IL-2. *J Immunol* **135**: 2865–2875

Matsuda T, Hirano T, Nagasawa S and Kishimoto T (1989) Identification of alpha2-macroglobulin as a carrier protein for IL-6. *J Immunol* **142**: 148–152

May LT, Viguet H, Kenney JS, Ida N, Allison AC and Sehgal PB (1992) High levels of 'complexed' interleukin-6 in human blood. *J Biol Chem* **267**: 19698–19704

May LT, Santhanam U and Sehgal PB (1994) On the multimeric nature of natural human interleukin-6. *J Biol Chem* **266**: 9950–9955

Meager A, Leung H and Woolley J (1989) Assays for tumour necrosis factor and related cytokines. *J Immunol Methods* **116**: 1–17

Meffert M, Schomburg A, Hänninen EL, Menzel T, Vocke S, Dallmann I, Grosse J, Duensing S, Buer J, Kirchner H, Poliwoda H and Atzpodien J (1995) In vivo tumor necrosis factor-alpha as indicator of biologic and clinical response to low-dose SC recombinant interleukin 2. *Anticancer Res* **15**: 127–132

Nadeau RW, Satoh H, Scheide S, Crowl R, Conroy R, Garland WA and Liberato DJ (1995) A comparison of mass balance, pharmacokinetics and disposition of [$^{14}C(U)$]- and [$^{125}I$]recombinant human interleukin 2 in cynomolgus monkeys. *Drug Metab Dispos* **23**: 904–909

Nakahama H, Tanaka Y, Shirai D, Miyazaki M, Imai N, Yokokawa T, Okada M and Kubori S (1992) Plasma interleukin-6 levels in continuous ambulatory peritoneal dialysis and hemodialysis patients. *Nephron* **61**: 132–134

Ohnishi H, Chao JTY, Lin KKM, Lee H and Chu TM (1989) Role of the kidney in metabolic change of interleukin-2. *Tumour Biol* **10**: 202–214

Ohnishi H, Lin KM and Chu TM (1990) Prolongation of serum half-life of interleukin 2 and augmentation of lymphokine-activated killer cell activity by pepstatin in mice. *Cancer Res* **50**: 1107–1112

Pepys MB (1981) C-reactive protein fifty years on. *Lancet* 21 March 653–656

Pereira BJ, Shapiro L, King AJ, Falagas ME, Strom JA and Dinarello CA (1994). Plasma levels of IL-1 beta, TNF alpha and their specific inhibitors in undialyzed chronic renal failure, CAPD and hemodialysis patients. *Kidney Int* **45**: 890–896

Stadler WM, Richards JM and Vogelzang NJ (1992) Serum interleukin-6 levels in metastatic renal cell cancer: correlation with survival but not an independent prognostic indicator. *J Natl Cancer Inst* **84**: 1835–1836

Suc E, Neuville S, Lacombe JL, Dumazer P, Tourani JM and Bugat R (1995) Interleukin-2 in a haemodialysis patient with metastatic renal cell cancer. *Presse Medicale* **24**: 327

Tartour E, Dorval T, Mosseri V, Deneux L, Mathiot C, Brailly H, Montero F, Joyeux I, Pouillart P and Fridman WH (1994) Serum interleukin 6 and C-reactive protein levels correlate with resistance to IL-2 therapy and poor survival in melanoma patients. *Br J Cancer* **69**: 911–913

Wadhwa M, Bird C, Page L, Mire-Sluis A and Thorpe R (1995) Quantitative biological assays for individual cytokines. In *Cytokines*: a *Practical Approach*, 2nd edn, Balkwill FR (ed.), pp. 357–391. IRL Press: Oxford

Ward RA, Gordon L (1993) Soluble tumor necrosis factor receptors are increased in hemodialysis patients. ASAIO *J* **39**: M782–6

Whittington R, Faulds D (1993) Interleukin-2: A review of its pharmacological properties and therapeutic use in patients with cancer. *Drugs* **46**: 446–514

© Cancer Research Campaign 1997